# Elevated CO_2_ and ammonium nitrogen promoted the plasticity of two maple in great lakes region by adjusting photosynthetic adaptation

**DOI:** 10.3389/fpls.2024.1367535

**Published:** 2024-04-09

**Authors:** Lei Wang, Qing-Lai Dang

**Affiliations:** ^1^ Jiyang College, Zhejiang A&F University, Zhuji, Zhejiang, China; ^2^ Faculty of Natural Resources Management, Lakehead University, Thunder Bay, ON, Canada

**Keywords:** global change, nitrogen form, amur maple, boxelder maple, photosynthetic adaptation

## Abstract

**Introduction:**

Climate change-related CO_2_ increases and different forms of nitrogen deposition are thought to affect the performance of plants, but their interactions have been poorly studied.

**Methods:**

This study investigated the responses of photosynthesis and growth in two invasive maple species, amur maple (*Acer ginnala* Maxim.) and boxelder maple (*Acer negundo* L.), to elevated CO_2_ (400 µmol mol^-1^ (aCO_2_) vs. 800 µmol mol^-1^ (eCO_2_) and different forms of nitrogen fertilization (100% nitrate, 100% ammonium, and an equal mix of the two) with pot experiment under controlled conditions.

**Results and discussion:**

The results showed that eCO_2_ significantly promoted photosynthesis, biomass, and stomatal conductance in both species. The biochemical limitation of photosynthesis was switched to RuBP regeneration (related to *J_max_*) under eCO_2_ from the Rubisco carboxylation limitation (related to *V_cmax_*) under aCO_2_. Both species maximized carbon gain by lower specific leaf area and higher N concentration than control treatment, indicating robust morphological plasticity. Ammonium was not conducive to growth under aCO_2_, but it significantly promoted biomass and photosynthesis under eCO_2_. When nitrate was the sole nitrogen source, eCO_2_ significantly reduced N assimilation and growth. The total leaf N per tree was significantly higher in boxelder maple than in amur maple, while the carbon and nitrogen ratio was significantly lower in boxelder maple than in amur maple, suggesting that boxelder maple leaf litter may be more favorable for faster nutrient cycling. The results suggest that increases in ammonium under future elevated CO_2_ will enhance the plasticity and adaptation of the two maple species.

## 1 Introduction

Global change can affect the structure and productivity of ecosystems via its effects on the physiological processes of individual plants (Campoy et al., 2021). Elevated atmospheric CO_2_, nitrogen (N) deposition and plant invasion are drivers of ecosystem changes (Bäurle et al., 2023; de Souza, 2023). A good understanding of plant phenotypic plasticity in response to elevated CO_2_ and N deposition is essential for predicting the survival and growth of plant species under the predicted future climate conditions (Du et al., 2019; Liu et al., 2022).

Studies have shown that elevated CO_2_ levels tend to have a fertilization effect on plants (Zhu et al., 2021)However, the increases in photosynthetic rate induced by elevated CO_2_ do not necessarily always lead to increased biomass accumulation and growth (Skinner et al., 2018; Cabon et al., 2022; Green and Keenan, 2022). Elevated CO_2_ can result in a downregulation of leaf photosynthetic capacity (maximum rate of ribulose-1,5-bisphosphate carboxylation, *V_cmax_*; maximum of photosynthetic electron transport rate, *J_max_*) and stomatal conductance (*g_s_*) (Sperry et al., 2019; Tcherkez et al., 2020). Photosynthetic downregulation under elevated CO_2_ is generally resulted from increases in photosynthetic carbohydrate production and subsequent dilution in leaf nitrogen concentration (Yin et al., 2019). Under elevated CO_2_, the limitation of photosynthesis In C3 plants can shift to RuBP regeneration (indicated by *J_max_*) from Rubisco carboxylation (indicated by *V_cmax_*) (Dusenge et al., 2019; Smith and Keenan, 2020) although co-limitation by the two processes is generally the norm in most C3 species (Smith and Keenan, 2020). Photosynthesis can also be limited by CO_2_ diffusions, such as *g_s_* and mesophyll conductance (*g_m_*) (Sakoda et al., 2021). The adaptability of invasive tree species under elevated CO_2_ should be fully explored.

Elevated CO_2_ can inhibit the absorption and assimilation of nitrate but magnify the effects of ammonium addition (Domiciano et al., 2020). Elevated CO_2_ may affect nitrate assimilation by inhibiting photorespiration because the assimilation process depends on reductants produced by photorespiration (Ainsworth and Long, 2021). Some studies suggest that elevated CO_2_ directly inhibits the activity of nitrate reductase (Wujeska-Klause et al., 2019). The relative abundance of ammonium and nitrate depends vary with soil type, microbial community, forest successional stage and atmospheric N deposition (Poucet et al., 2021; Wang et al., 2023). Ammonium is generally the main nitrogen source in late successional stages (Bloom, 2015). With the increasing proportion of ammonium in atmospheric N deposition, N source forms become more important in the study of plant response to climate change (Luo et al., 2022).

Plant invasion is also a driver of changes in plant communities associated with global change (Petruzzellis et al., 2021). Invasive plants can benefit more from elevated CO_2_ than local species and hence climate change can further promote plant invasion and changes in species composition (Rathee et al., 2021). Plants usually have higher phenotypic plasticity in photosynthesis and biomass allocation, especially in key leaf traits (Specific leaf area (*SLA*), *N_area_*, and net photosynthesis rate (*A_n_*)) (Onoda et al., 2017; Liu et al., 2022). Invasive plants also have higher capability for resource capture and utilization efficiency by reducing the cost of leaf construction (Barros et al., 2020). Plant invasion threatens biodiversity and ecosystem services (Petruzzellis et al., 2021). However, invasive plants can also lead to soil eutrophication and improve vegetation productivity (Lee et al., 2017). These paradoxes make it difficult to predict the response of plants to future global changes, especially elevated CO_2_ and different forms of N deposition. It has been found that increases in ammonium in the soil are conducive to plant invasion, but its interaction with elevated CO_2_ is poorly understood (Chen et al., 2021).

Amur maple (*Acer ginnala* Maxim.) is native tree species in northeast Asia and is popular as its leaves produce antioxidants similar to that of green tea (Bi et al., 2016). Amur maple has high ornamental value and is widely introduced in North America in the 1960s. Due to its tenacious adaptability, amur maple is listed as an invasive plant species by the Natural Resources Conservation Service and the Forestry Service of the United States Department of Agriculture (USDA, 2005). Boxelder maple (A. *negundo* L.) is a native pioneer tree species in North America with strong adaptability and has successfully invaded Europe, Asia, South America and Australia (Saccone et al., 2010; McEvoy et al., 2022). Boxelder maple has strong phenotypic plasticity and resource allocation ability but can promote the invasion by other species because of the fast nitrogen cycling of its litter (Porté et al., 2011). A recent genomic study suggests that boxelder maple has a smaller genome with recent gene family evolution which might be related to tendencies (McEvoy et al., 2022). However, there are few studies on the responses of these two invasive maple species to elevated CO_2_ and N forms. T The purpose of this study was to verify the following scientific questions: 1. Elevated CO_2_ promoted the photosynthetic rate of two maple trees and changed the biochemical limits of photosynthesis; 2. The morphological plasticity of leaves was sensitive to climate change; 3. Compared with nitrate, ammonium nitrogen was conducive to the growth of two maple under elevated CO_2_ condition.

## 2 Materials and methods

### 2.1 Plant materials and treatments

The seeds of Amur maple and boxelder maple were collected from 6 mature trees at least 50 m apart from each other in the natural forests (amur maple at 48.416N, 89.267W; boxelder maple at 48.429N, 89.261W) near Lakehead University Thunder Bay campus (Ontario, Canada). The seeds were stratified in wet sand at 4°C for about 60 days before being sown in germination trays. Seedlings with 10 cm high were transplanted into 4 L plastic pots filled with a mixture of vermiculite and peat moss (1:1, v:v) (Sun Gro^®^, 770 Silver Street, Agawam, MA, USA) and were treated with different CO_2_ and nitrogen form.

The experiment was conducted in four research greenhouses (G1, G2, G3, G4 see **Table 1**) on the Thunder Bay campus of Lakehead University. The experiment followed a split plot design with two [CO_2_] levels [ambient 400 µmol mol^-1^ (aCO_2_) and elevated 800 µmol mol^-1^ (eCO_2_)] as the main plot, and three nitrogen treatments as the split plot (10 mM ammonium, 10 mM nitrate, 10 mM N with equal proportion of ammonium and nitrate) (**Table 1**). The two tree species were nested within the N-CO_2_ combination. Each CO_2_ level had two replicates (two separate greenhouses). Each replicate of each treatment combination had 8 seedlings of each species (a total of 96 seedlings per species: 2 CO_2_ * 2 replicates * 3 N forms * 8 seedlings).

**Table 1:** Outline of the experimental design and treatments.

Treatments	CO_2_ (µmol mol^-1^)	Ammonium (mM)	Nitrate (mM)	Greenhouse	Amur maple (seedlings)	Boxelder maple (seedlings)
1	400	10		G1	8	8
1	400	10		G2	8	8
2	400	5	5	G1	8	8
2	400	5	5	G2	8	8
3	400		10	G1	8	8
3	400		10	G2	8	8
4	800	10		G3	8	8
4	800	10		G4	8	8
5	800	5	5	G3	**8**	8
5	800	5	5	G4	8	8
6	800		10	G3	8	8
6	800		10	G4	**8**	8

Nitrogen treatments are three levels of 10 mM ammonium, 5 mM ammonium & 5 mM nitrate, 10 mM nitrate. G1 and G2 are the two replicates greenhouse with ambient 400 µmol mol^-1^ CO_2_, G3 and G4 represent two replicates greenhouse with 800 µmol mol^-1^ elevated CO_2_.

The nitrogen sources used ammonium-sulfonate (NH_4_)_2_SO_4_ for ammonium and sodium-nitrate NaNO_3_ for nitrate (BioBasic Inc. 20 Konrad Crescent, Markham, ON, Canada). All N treatments received identical amounts of other nutrient elements: 5 mM Potassium phosphate and 4 g L^-1^ Micromax Micronutrients Granular (Calcium 6%, Magnesium 3%, Sulphur 12%, Boron 0.1%, Copper 1%, Iron 17%, Manganese 2.5%, Molybdenum 0.05%, Zinc 1%) (Everris NA Inc. P.O. Box 3310, Dublin, OH, USA). The extra S provided with the ammonium formulation and the extra Na provided with the nitrate formulation were compensated in the nutrient solution to avoid differences in other elements between the different N treatments. The seedlings were fertilized twice a week and irrigated with 500 ml water within fertilizer according to the corresponding treatment every other day. The seedlings were randomly changed the position within the same greenhouse in each treatment every two weeks.

The CO_2_ concentration in each greenhouse was maintained using by a CO_2_ generator (GEN-2E, Custom Automated Products Inc., Riverside, California, USA). Other environmental conditions in all the greenhouses were 25/16°C (day/night) temperature, 50% RH and 16-h photoperiod. High-pressure sodium lamps (P.L. Systems, Grimsby, ON, Canada) were used to supplement then natural light when the ambient light intensity was below 500 µmol m^-2^ s^-1^ or the natural daylength was shorter than 16-h. All the environmental variables in each greenhouse were monitored and controlled by an Argus Titan Environment System (Argus Control Systems Ltd. Vancouver, BC, Canada).

### 2.2 Gas exchange and pigment measurement

After three months treatments, six seedlings were randomly selected from each treatment combination and foliar gas exchange was measured between 9:00 am and 16:00 pm on a unshaded mature leaf on the near the top of the canopy using a LI-6800 Portable Photosynthesis System (LI-COR Biosciences, 4647 Superior Street, Lincoln, Nebraska, USA). The gas exchange under treatment CO_2_ was measured at the corresponding growth [CO_2_] (aCO_2_ at 400 µmol mol^-1^, eCO_2_ at 800 µmol mol^-1^), 25°C temperature, 1.1 - 1.3 kPa water vapor pressure deficit, 1000 µmol m^-2^ s^-1^ photosynthetically active radiation flux density. At least 30 minutes was allowed before a steady state reading was taken. The net photosynthetic rate (*A_n-g_*), stomatal conductance (*g_s_*), intercellular [CO_2_] to ambient [CO_2_] ratio (*C_i_*/*C_a_*), and photosynthetic nitrogen uses efficiency (*PNUE* = *A_n-g_*/*N_area_*) were subjected to statistical analyses.

Photosynthetic response to intercellular [CO_2_] (*A*/*C_i_*) curves were measured at 400, 300, 200, 100, 50, 400, 500, 600, 800, 1000, 1200, 1500 µmol mol^-1^ [CO_2_], 25°C temperature, 1.1 - 1.3 kPa VPD, and 1000 µmol m^-2^ s^-1^ PAR. The maximum rate of Rubisco carboxylation *V_cmax_* and maximum rate of photosynthetic electron transport *J_max_* were estimated using the fitaci function, and the transition point (*C_i-t_*, *A_n-t_*) of biochemical limitation from Rubisco to RuBP regeneration was estimated using findCiTranstion function in the Plantecophys R package from *A*/*Ci* data (Duursma, 2015). The initial slope of *A*/*C_i_* was estimated as apparent carboxylation efficiency (*ACE*), and the X-axis intercept was evaluated as the CO_2_ compensation point (*Γ_ACi_*).

**Figure 1:**
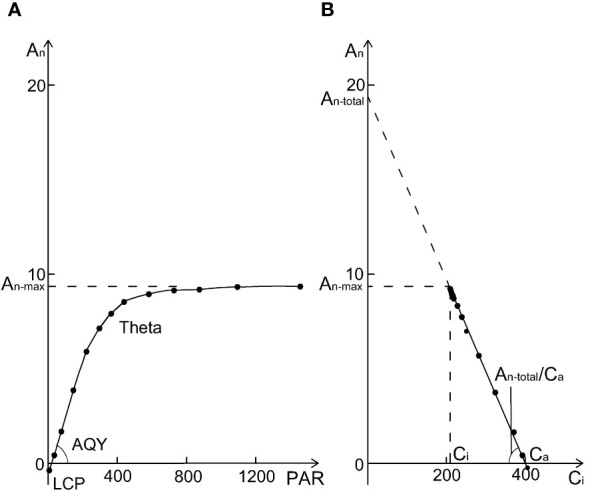
A sample of a photosynthetic light response curve (lrc) with relevant parameters **(A)** the photosynthetic rate at saturation light and 400 µmol mol^−1^ CO_2_ (*A_n-max_*), the curvature of the lrc (Theta, θ), apparent quantum yield (the initial slope of lrc, *AQY*), and light compensation point (*LCP*). *A_n_* vs. *C_i_* derived from the lrc database **(B)**, where *A_n-total_* and *A_n-total/_C_a_* are the y-intercept and slope of the *A_n_* - *C_i_* regression line. From **(B)**, *A_n-total_*/*C_a_* = *A_n-max_*_/_(*C_a_* - *C_i_*), so *A_n-max_*/*A_n-total_* = (*C_a_* - *C_i_*)/*C_a_*, which means that *A_n-max_*/*A_n-total_* + *C_i_*/*C_a_* = 1.

The photosynthetic light response curve (lrc) was measured at 400 µmol mol^-1^ [CO_2_] and 1000, 1500, 1200, 900, 600, 300, 150, 50, 0 µmol m^-2^ s^-1^ PAR (**Figure 1A**). Other measurement environment conditions were the same as *A*/*C_i_* curve measurement. Curvature (*θ*) and photosynthetic rate of saturation light (*A_n-max_*) were fitted from the lrc data by non-rectangular hyperbola model using non-linear least squares in R (Salter et al., 2019). The initial slope of the lrc was estimated as the apparent quantum yield (*AQY*) and the X-axis intercept was estimated as the light compensation point (*LCP*). The fitting line of *A_n_* vs. *C_i_* from lrc dataset (**Figure 1B**) was used to estimate *A_n-total_* by the Y-axis intercept and *A_n-total_*/*C_a_* as the initial slope (Wang and Dang, 2023).

The initial line portions of three *A*/*C_i_* were measured with 200, 150, 100, and 50 µmol mol^-1^ of [CO_2_], and 300, 150, and 75 µmol m^-2^ s^-1^ PAR, from which the daytime respiratory rate (*R_d_*) and intercellular CO_2_ compensation point (*C_i_* *) were calculated using Walker’s slope intercept method (Walker et al., 2016). The variable *J* method was employed to calculate mesophyll conductance (*g_m_*) (Harley et al., 1992), using the equation Γ* = *C_i_* * + *R_d_*/*g_m_* ( (Walker et al., 2016), where Γ* is the CO_2_ compensation point in absence of day respiration.

After the gas exchange measurement, the tested leaves were immediately removed and stored in a refrigerator (-70°C). Leaf pigment was extracted using 80% acetone and the absorbance at 645 nm and 663 nm measured. Chlorophyll concentration (*Chl*) of the leaves was calculated with *Chl* = 20.2 * A645 + 8.02 * A663 (Wellburn, 1994).

### 2.3 Relative photosynthetic limitation and nitrogen partitioning

The relative limitations of photosynthesis by biochemical (*l_b_*), mesophyll conductance (*l_m_*) and stomatal conductance (*l_s_*) were calculated according to Grassi and Magnani (Grassi and Magnani, 2005): *l_b_* = *g_t_*/(*g_t_* + *әA_n_*/*әC_c_*); *l_s_* = (*g_t_/g_s_* * *әA_n_*/*әC_c_*)/(*g_t_* + *әA_n_*/*әC_c_*); *l_m_* = (*g_t_/g_m_* * *әA_n_*/*әC_c_*)/(*g_t_* + *әA_n_*/*әC_c_*), where *g_t_* is the total conductivity of CO_2_ diffusion (*g_t_* = 1/(1/*g_m_* + 1/*g_s_*)), *әA_n_*/*әC_c_* is the initial slope of the *A_n_* to *C_c_* response curve and was calculated using the equation *әA_n_/әC_c_* = *V_cmax_*/(Γ* + *K_m_*) (Farquhar et al., 1980), where Γ* was as noted previously and *K_m_* was calculated by *K_m_* = *K_c_* (1 + O/*K_o_*) (Bernacchi et al., 2001).

We investigated the partitioning of leaf N into carboxylation (*N_cb_*), electron transfer (*N_et_*), light capture (*N_lc_*) and respiration (*N_resp_*). The calculation is based on Trouwborst’s method (Trouwborst et al., 2011).

### 2.4 Biomass and nitrogen measurement

After three months CO_2_ and nitrogen form treatments, the height of all the seedlings was measured. Leaf area was measured using a Regent WinFolia system (Regent Instruments Inc., Quebec City, QC, Canada). The seedlings were then harvested and separated into roots, stems, and leaves, and oven-dried at 75°C for 48 hours to constant weight to determine the biomass of different organs and specific leaf area (*SLA*). Leaf mass-based N concentration (*N_mass_*) and carbon concentration (C) were measured using the dry combustion method on a CNS-2000 (LECO Corp., St. Joseph, MI, USA) at the Lakehead University Centre for Analytical Services. Area-based leaf N concentration (*N_area_*) and total leaf N content per plant (*N_leaf_*) were determined from leaf biomass and *SLA*.

### 2.5 Statistical analysis

The effects of CO_2_ and N forms on photosynthetic and growth traits of individual tree species were investigated using two-way analysis of variance using the agricolae package in R. A three-way (CO_2_, N forms and species) ANOVA was performed for *A_n-g_* and *PNUE*. Logarithmic or power transformation was used to transform variables that did not meet the normality and homogeneity assumptions before ANOVA was carried out. When a interaction was statistically significant, Tukey *post hoc* pairwise comparisons were conducted for the means. The principal component analysis (PCA) function of the FactoMineR package was used for PCA to investigate the relationship and clustering of physiological and growth-related parameters. The Bowen method (Bowen et al., 2017) was used to investigate the direct/indirect effects of CO_2_ and N sources as categorical variables on biomass and *A_n-g_* using the structural equation model (SEM) and the psem function in the pieceweSEM package (Lefcheck, 2016). Based on the leaf economic spectroscopy (LES) and a prior structural model (Onoda et al., 2017; Liu et al., 2022), biomass was explained by *A_n-g_*, *N_area_* and *SLA*, and *A_n-g_* was explained by photosynthetic capacity, *Chl* and *g_t_*. The photosynthetic capacity was obtained by the regression equations from *V_cmax_* and *J_max_*. All the statistical analyses were performed using R.

## 3 Results

### 3.1 Growth in response CO_2_ and N form

**Figure 2:**
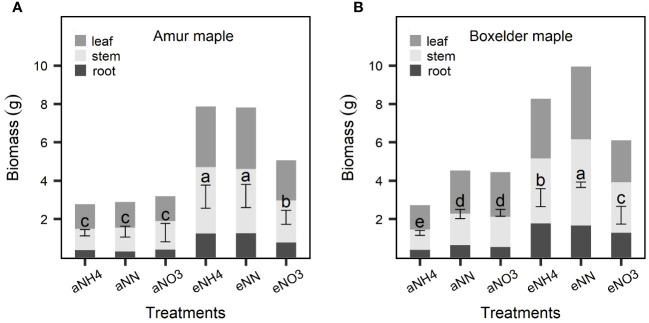
Biomass responses to CO_2_ and N form treatments in amur maple **(A)** and boxelder maple **(B)**. “a-” means ambient CO_2_ (400 µmol mol^−1^) and “e-” means elevated CO_2_ (800 µmol mol^−1^) treatments. NH4: fertilized 10 mM (NH_4_)_2_SO_4_; NN: fertilized with a combination of 5 mM (NH_4_)_2_SO_4_ and 5 mM NaNO_3_; NO3: fertilized with 10 mM NaNO_3_. Means (± SE, n=6) with different letters indicated significant differences between treatments (Tukey host hoc test, P<0.05).

The eCO_2_ significantly increased the biomass of both maple species but the effect was much bigger seedlings fertilized with NH4 or a combination of NH4 and NO3 than those fertilized with NO3 only (**Figure 2**). While the general response patterns were similar in the two species, there were differences in the responses between the two species: under the aCO_2_, no significant effect of N form on the biomass was observed in amur maple (**Figure 2A**) but it was significantly lower in the boxelder maple seedlings that were supplied with ammonium only than those of seedlings that were fertilized with the other two N treatments (**Figure 2B**); Under eCO_2_ treatment, the nitrate nitrogen only treatment significantly decreased the biomass of both maple species (**Figure 2**). CO_2_ treatment significantly affected seedling height and leaf area (**Tables 1**, **3**). However, these values of the boxelder maple were influenced by the interaction of the two treatments and showed a trend similar to that of biomass (**Table 3**). In general, the eCO_2_ treatment significantly reduced *SLA*, except for amur maple treated with nitrate and boxelder maple treated with ammonium (**Tables 2**, **3**).

**Table 2:** Height, leaf area, specific leaf area (*SLA*), and nitrogen-related traits of amur maple seedlings at ambient and elevated CO_2_ in response to different forms of N supply.

CO_2_	N	Hight (cm)	Leaf area (cm^-2^)	*SLA* (cm^2^ g^-1^)	*N_area_* (g m^-2^)	*N_leaf_* (mg plant^-1^)	C/N
aCO_2_	NH4	60.5 ± 3.1 c	286 ± 19 c	225 ± 6 a	1.1 ± 0.05 b	30.3 ± 3 c	19.9 ± 0.6 ab
	NN	74.5 ± 6 b	312 ± 35 c	233 ± 4 a	1.2 ± 0.03 ab	37.2 ± 4.2 bc	16.3 ± 0.4 c
	NO3	76.8 ± 4.8 b	301 ± 50 c	231 ± 5 a	1 ± 0.04 b	30.5 ± 4.8 c	19.2 ± 0.7 ab
eCO_2_	NH4	95.8 ± 2.2 a	654 ± 60 a	206 ± 4 b	1.3 ± 0.06 a	86.7 ± 11.7 a	17.5 ± 1 bc
	NN	95.5 ± 3.8 a	614 ± 55 a	192 ± 7 b	1.2 ± 0.08 ab	75 ± 9.1 a	20.2 ± 1.4 a
	NO3	93 ± 3.9 a	465 ± 32 b	223 ± 5 a	1.2 ± 0.04 ab	54.3 ± 5.3 b	17.7 ± 0.7 bc
*P*-value	CO_2_	**< 0.001**	**< 0.001**	**< 0.001**	**0.005**	**< 0.001**	0.994
	N	0.187	0.11	**0.02**	0.127	0.063	0.905
	CO_2_: N	0.073	0.079	**0.01**	0.107	0.084	**0.002**

each value represents mean ± SE (n=6). Two-way ANOVA was performed to analyze CO_2_ and nitrogen (N) as well as their interactive effects (CO_2_: N). Significant effects (P ≤ 0.05) are marked in bold and “:” indicated interaction. Different letters within the same column indicated statistically significant differences between treatments (Tukey post hoc test, P<0.05). aCO_2_: ambient CO_2_ (400 µmol mol^−1^); eCO_2_: elevated CO_2_ (800 µmol mol^−1^); NH4: fertilized 10 mM (NH_4_)_2_SO_4_; NN: fertilized 10 mM N with 5 mM (NH_4_)_2_SO_4_ and 5 mM NaNO_3_; NO3: fertilized 10 mM NaNO_3_. SLA: specific leaf area; N_area_: leaf N content based on leaf area; N_leaf_: whole plant leaf N content; C/N: leaf carbon-nitrogen ratio.

**Table 3:** Hight, leaf area, specific leaf aera (SLA), and nitrogen-related traits of boxelder maple grown at ambient and elevated CO_2_ response different forms of N supply.

CO_2_	N	Hight (cm)	Leaf area (cm^-2^)	*SLA* (cm^2^ g^-1^)	*N_area_* (g m^-2^)	*N_leaf_* (mg plant^-1^)	C/N
aCO_2_	NH4	45 ± 1.4 c	836 ± 74 d	600 ± 5.1 a	0.6 ± 0.05 b	52.1 ± 4.2 c	10.6 ± 0.2 b
	NN	52.8 ± 2.3 bc	1081 ± 91 cd	523 ± 11.5 bc	1 ± 0.09 a	106.1 ± 9.3 ab	8.9 ± 0.3 c
	NO3	56.2 ± 1.4 b	1293 ± 70 bc	557 ± 14.5 ab	0.8 ± 0.05 ab	104.1 ± 6.9 ab	9.2 ± 0.3 c
eCO_2_	NH4	70.7 ± 0.9 a	1435 ± 87 ab	518 ± 14 bc	0.9 ± 0.09 ab	123.6 ± 16.2 a	11.1 ± 0.6 ab
	NN	76 ± 1.8 a	1625 ± 54 a	438 ± 7.7 d	0.9 ± 0.04 ab	145.1 ± 4.3 a	11.1 ± 0.3 ab
	NO3	58.7 ± 2.8 b	1064 ± 127 cd	483 ± 8.6 cd	0.8 ± 0.17 ab	81 ± 11.2 bc	11.9 ± 0.7 a
*P*-value	CO_2_	**< 0.001**	**< 0.001**	**< 0.001**	0.565	**< 0.001**	**< 0.001**
	N	**< 0.001**	**0.042**	**< 0.001**	0.111	**< 0.001**	0.18
	CO_2_: N	**< 0.001**	**< 0.001**	0.874	0.22	**< 0.001**	**0.046**

each value represents mean ± SE (n=6). Two-way ANOVA was performed to analyze CO_2_ and nitrogen (N) as well as their interactive effects (CO_2_: N). Significant effects (P ≤ 0.05) are shown in bold and “:” indicated interaction. Different letters within the same column indicated statistically significant differences between treatments (Tukey post hoc test, P<0.05). aCO_2_: ambient CO_2_ (400 µmol mol^−1^); eCO_2_: elevated CO_2_ (800 µmol mol^−1^); NH4: fertilized 10 mM (NH_4_)_2_SO_4_; NN: fertilized 10 mM N with 5 mM (NH_4_)_2_SO_4_ and 5 mM NaNO_3_; NO3: fertilized 10 mM NaNO_3_. SLA: specific leaf area; N_area_: leaf N content based on leaf-area; N_leaf_: whole plant leaf N content; C/N: leaf carbon-nitrogen ratio.

### 3.2 Leaf nitrogen and PNUE

Treatment of eCO_2_ significantly increased *N_area_* in amur maple (**Table 2**) but not in boxelder maple (**Table 3**). In general, eCO_2_ treatment promoted the *N_leaf_* of the two tree species with the except of boxelder maple fertilized with nitrate (**Table 3**). The eCO_2_ treatment significantly promoted the leaf C/N value of boxelder maple (**Table 3**) but not of amur maple (**Table 2**).

**Figure 3:**
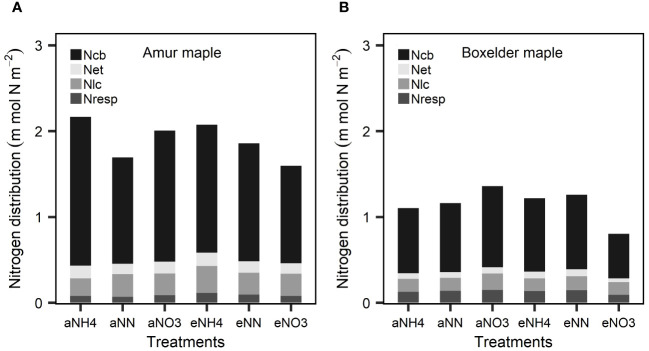
Leaf N partitioning into carboxylation (*N_cb_*), electron transfer (*N_et_*), light capture systems (*N_lc_*), and respiratory (*N_resp_*) in response to CO_2_ and different N forms in amur maple **(A)** and boxelder maple **(B)**. “a-” means ambient CO_2_ (400 µmol mol^−1^) and “e-” means elevated CO_2_ (800 µmol mol^−1^) treatments. NH4: fertilized with 10 mM (NH_4_)_2_SO_4_; NN: fertilized with a combination of 5 mM (NH_4_)_2_SO_4_ and 5 mM NaNO_3_; NO3: fertilized with 10 mM NaNO_3_.

**Figure 4:**
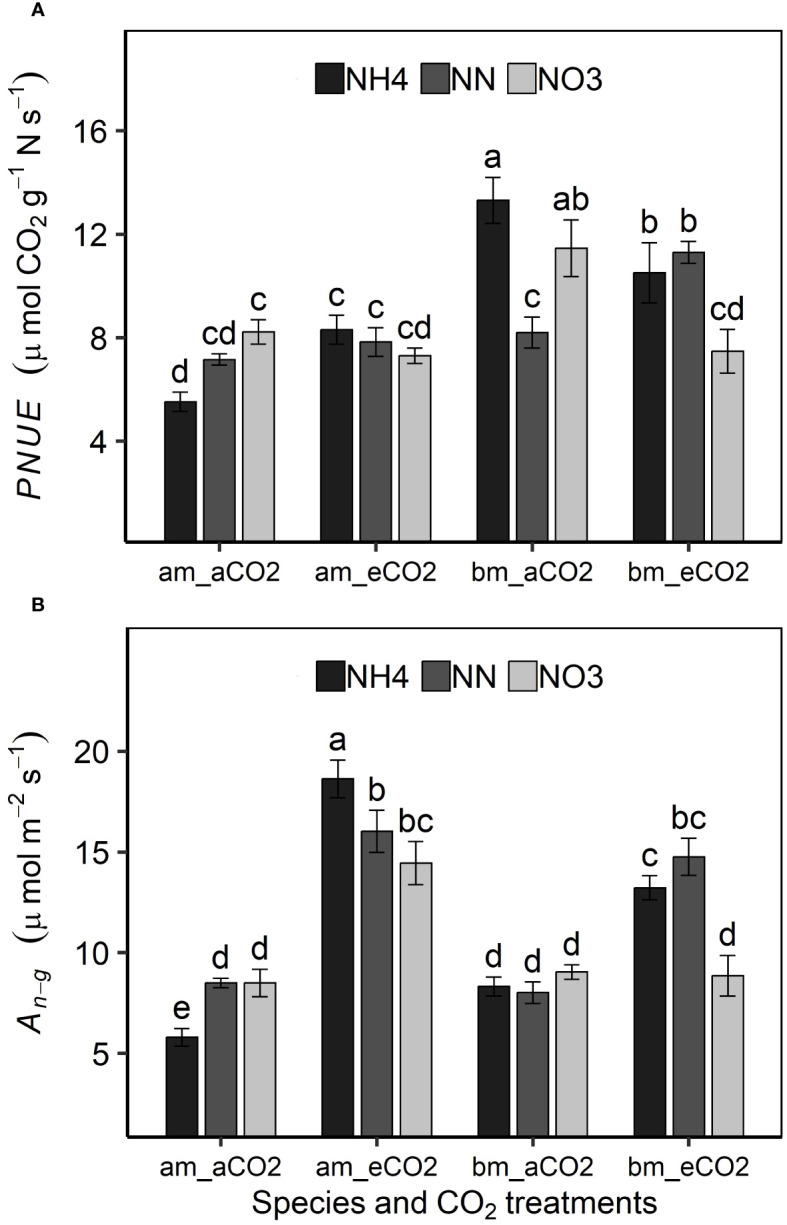
Effects of CO_2_ and N forms on photosynthetic nitrogen use efficiency [*PNUE*, (**A**) and photosynthetic rate at growth CO_2_ (*A_n-g_*, (**B**)] in amur maple (am) and boxelder maple (bm). aCO_2_: ambient CO_2_ (400 µmol mol^−1^); eCO_2_: elevated CO_2_ (800 µmol mol^−1^); NH4: fertilized with 10 mM (NH_4_)_2_SO_4_; NN: fertilized with a combination of 5 mM (NH_4_)_2_SO_4_ and 5 mM NaNO_3_; NO3: fertilized with 10 mM NaNO_3_. Means (± SE, n=6) with different letters indicated significant differences between treatments (Tukey host hoc test, P<0.05).

Amur maple distributed relatively larger proportions of leaf N to *N_cb_*, Net and *N_lc_* than did of boxelder maple while N allocation to *N_resp_* was larger in boxelder maple than amur maple (**Figure 3**). Nitrate and eCO_2_ treatment slightly limited leaf N allocation to carboxylation in both species (**Figure 2**). In general, the *PNUE* of boxelder maple was higher than that of amur maple, except for NN treatment under aCO_2_ and nitrate treatment under eCO_2_ (**Figure 4A**). Ammonium significantly reduced *PNUE* in amur maple under aCO_2_ treatment (**Figure 4A**).

### 3.3 Photosynthesis traits

*A_n-g_* was promoted by eCO_2_ and its response in both tree species was similar to that of biomass under different treatments (**Figure 4B**). The promoting effect of eCO_2_ on *A_n-g_* was smaller or not statistically significant under nitrate treatment (**Figure 4B**). Nitrate decreased both *V_cmax_* and *J_max_* in both species under eCO_2_ although the effect was not always statistically significant but did not significantly affect either variable in either species aCO_2_ treatment (**Tables 4**, **5**). Ammonium significantly increased leaf *Chl* concentration of amur maple grown under eCO_2_ (**Table 4**) while nitrate significantly increased leaf *Chl* of boxelder maple grown under aCO_2_ (**Table 5**).

**Table 4:** Photosynthetic capacity, chlorophyll concentration, conductance to CO_2_ diffusion, and intercellular to external CO_2_ concentration ratio in amur maple seedlings grown at ambient or elevated CO_2_ in response to different forms of N source.

CO_2_	N	*V_cmax_* (µ mol m^-2^ s^-1^)	*J_max_* (µ mol m^-2^ s^-1^)	*Chl* (mg m^-2^)	*g_m_* (mol m^-2^ s^-1^)	*g_s_* (mmol m^-2^ s^-1^)	*C_i_*/*C_a_*
aCO_2_	NH4	73.1 ± 8.6 a	114 ± 6.1 b	412 ± 51 b	0.11 ± 0.01 a	77 ± 12.9 c	0.64 ± 0.03 ab
	NN	56.6 ± 1.2 bc	108 ± 3.3 b	507 ± 25 ab	0.07 ± 0.01 b	119 ± 4.2 bc	0.66 ± 0.01 ab
	NO3	69.2 ± 3.6 ab	119 ± 3.5 b	485 ± 36 b	0.05 ± 0 c	119 ± 9.6 bc	0.6 ± 0.02 b
eCO_2_	NH4	69.5 ± 1.9 a	140 ± 7.2 a	601 ± 24 a	0.04 ± 0 c	174 ± 12 a	0.73 ± 0.02 a
	NN	63.2 ± 3.1 ab	123 ± 8.5 ab	485 ± 37 b	0.04 ± 0.01 c	141 ± 23.7 ab	0.69 ± 0.04 ab
	NO3	48 ± 3.6 c	108 ± 8.4 b	497 ± 38 ab	0.08 ± 0.01 b	119 ± 20.8 bc	0.69 ± 0.03 ab
*P*-value	CO_2_	0.099	0.067	0.053	**< 0.001**	**0.004**	**0.003**
	N	**0.013**	0.1	0.912	0.133	0.758	0.301
	CO_2_: N	**0.0117**	**0.027**	**0.014**	**< 0.001**	**0.009**	0.396

each value represents mean ± SE (n=6). Two-way ANOVA was performed to analyze CO_2_ and nitrogen form (N) as well as their interactive effects (CO_2_: N). Significant effects (P ≤ 0.05) are marked in bold and “:” indicated interaction. Different letters within the same column indicated statistically significant differences between treatments (Tukey post hoc test, P<0.05). aCO_2_: ambient CO_2_ (400 µmol mol^−1^); eCO2: elevated CO_2_ (800 µmol mol^−1^); NH4: fertilized 10 mM (NH_4_)_2_SO_4_; NN: fertilized 10 mM N with 5 mM (NH_4_)_2_SO_4_ and 5 mM NaNO_3_; NO3: fertilized 10 mM NaNO_3_. V_cmax_: maximum rate of ribulose-1,5-bisphosphate carboxylation; J_max_: maximum photosynthetic electron transport rate; Chl: leaf chlorophyll concentration. g_m_: mesophyll conductance; g_s_: stomatal conductance; C_i_/C_a_: intercellular (C_i_) to ambient (C_a_) CO_2_ concentration ratio.

**Table 5:** Photosynthesis capacity, chlorophyll concentration, and CO_2_ diffusion conductance in boxelder maple seedlings grown at ambient and elevated CO_2_ in response to different forms of N source.

CO_2_	N	*V_cmax_* (µ mol m^-2^ s^-1^)	*J_max_* (µ mol m^-2^ s^-1^)	*Chl* (mg m^-2^)	*g_m_* (mol m^-2^ s^-1^)	*g_s_* (mmol m^-2^ s^-1^)	*C_i_*/*C_a_*
aCO_2_	NH4	34.9 ± 1.6 b	60.1 ± 1.9 ab	306 ± 21 b	0.09 ± 0.007 bc	60.2 ± 4.4 cd	0.64 ± 0.01 b
	NN	37.5 ± 1.8 b	59.7 ± 1.5 ab	297 ± 15 b	0.11 ± 0.004 bc	76.1 ± 7.1 ab	0.68 ± 0.02 b
	NO3	44 ± 1.1 a	67.2 ± 3.3 a	363 ± 21 a	0.09 ± 0.003 c	64.5 ± 4.5 cd	0.61 ± 0.02 b
eCO_2_	NH4	41.1 ± 0.8 ab	68.9 ± 3.4 a	306 ± 21 b	0.18 ± 0.017 a	84 ± 7.2 ab	0.78 ± 0.02 a
	NN	36 ± 3.4 b	66.4 ± 5.6 a	333 ± 14 ab	0.09 ± 0.004 c	89.7 ± 10.5 a	0.78 ± 0.02 a
	NO3	27.7 ± 3.1 c	47.8 ± 5.8 b	286 ± 14 b	0.12 ± 0.012 b	49.7 ± 6.6 d	0.7 ± 0.03 ab
*P*-value	CO_2_	**0.038**	0.692	0.365	**< 0.001**	0.199	**< 0.001**
	N	0.622	0.187	0.6	**< 0.001**	**0.004**	**0.004**
	CO_2_: N	**< 0.001**	**0.002**	**0.013**	**< 0.001**	**0.027**	0.43

each value represents mean ± SE (n=6). Two-way ANOVA was performed to analyze CO_2_ and nitrogen (N) as well as their interactive effects (CO_2_: N). Significant effects (P ≤ 0.05) are shown in bold and “:” indicated interaction. Different letters within the same column indicated statistically significant differences between treatments (Tukey post hoc test, P<0.05). aCO_2_: ambient CO_2_ (400 µmol mol^−1^); eCO_2_: elevated CO_2_ (800 µmol mol^−1^); NH4: fertilized 10 mM (NH_4_)_2_SO_4_; NN: fertilized 10 mM N with 5 mM (NH_4_)_2_SO_4_ and 5 mM NaNO_3_; NO3: fertilized 10 mM NaNO_3_. V_cmax_: maximum rate of ribulose-1,5-bisphosphate carboxylation; J_max_: maximum photosynthetic electron transport rate; Chl: leaf chlorophyll concentration. g_m_: mesophyll conductance; g_s_: stomatal conductance; C_i_/C_a_: intercellular (C_i_) to ambient (C_a_) CO_2_ concentration ratio.

Nitrate significantly decreased *g_m_* of amur maple under aCO_2_ condition but increased it under eCO_2_ (**Table 4**). In contrast, the *g_m_* of boxelder maple was intermediate in seedlings fertilized with nitrate under eCO_2_ while there was not significant difference among N treatments under aCO_2_ (**Table 5**). *C_i_*/*C_a_* was generally higher in eCO_2_ than under cCO_2,_ especially in boxelder maple (**Tables 4**, **5**).

### 3.4 A/C_i_ traits and photosynthetic limitations

**Figure 5:**
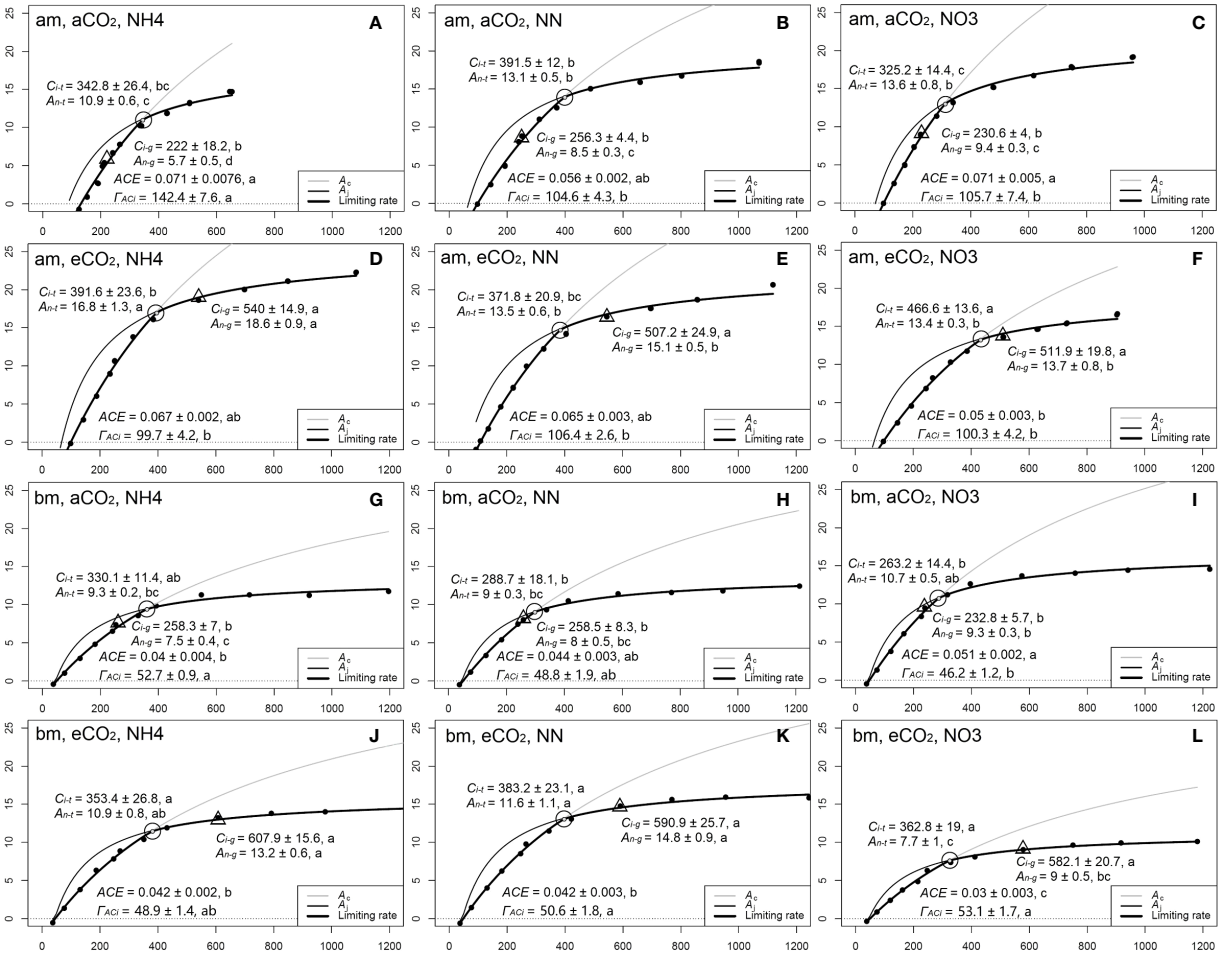
The A/Ci curves in response to different N forms in amur maple (am, **A–F**) and boxelder (bm, **G–L**) seedlings grown under ambient CO2 (aCO2, A-C in am and G-I in bm) and elevated CO2 (eCO2, **D–F** in am and **J–L** in bm). NH4: fertilized with 10 mM (NH4)2SO4 (left column); NN: fertilized with a combination of 5 mM (NH4)2SO4 and 5 mM NaNO3 (middle column); NO3: fertilized with 10 mM NaNO3 (right column). Each point denotes the means of six (*C_i_*, *A_n_*) values in *A*/*C_i_* curve. The circles represent the transition point (*C_i-t_*, *A_n-t_*) from Rubisco carboxylation to RuBP regeneration of photosynthesis limitation. The triangles indicated the photosynthetic rate (*C_i-g_*, *A_n-g_*) under growth *C_a_* (400 µmol mol^−1^ versus 800 µmol mol^−1^). *ACE*: apparent carboxylation efficiency estimated from the initial slope of *A*/*C_i_* curve; *Γ_ACi_*: CO_2_ compensation point estimated from *A*/*C_i_* curve intersects point on *X*-axis. Different letters of the same parameter in the same species are significantly different between treatments (Tukey host hoc test, P<0.05, see **Supplementary Table S2**).

The transition point (*C_i-t_*, *A_n-t_*) of photosynthetic limitation from Rubisco carboxylation to RuBP regeneration ion shifted to higher *C_i-g_* and higher *A_n-g_* under eCO_2_ in both species (**Figure 5**). The transition point was much lower in amur maple seedlings fertilized with ammonium than other N treatments under aCO_2_, but *Γ_ACi_* was greater (**Figures 5A–C**). Ammonium significantly promoted (*C_i-g_*, *A_n-g_*) and (*C_i-t_*, *A_n-t_*) in amur maple (**Figures 5D–F**), nitrate lowered the transition point in boxelder maple under eCO_2_ (**Figures 5J–L**).

**Figure 6:**
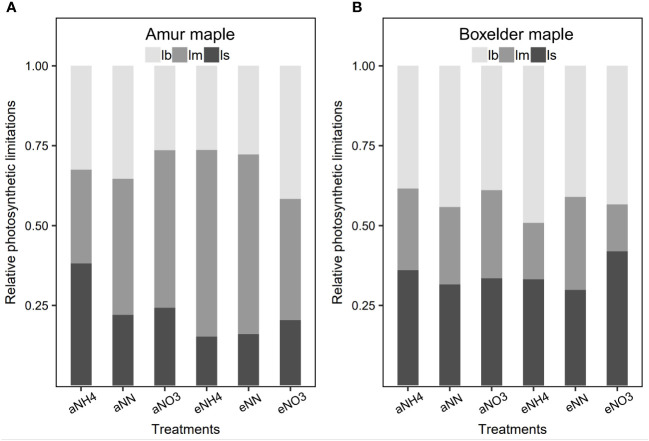
The relative photosynthesis limitations of biochemistry (*l_b_*), stomatal resistance (*l_s_*), and mesophyll resistance (*l_m_*) in response to CO_2_ and N form treatment in amur maple **(A)** and boxelder maple **(B)**. Abbreviations are provided in **Supplementary Table S1**.

Relative photosynthetic limitation analysis showed that photosynthesis was primarily limited by *g_s_* in amur maple seedlings treated with aCO_2_ and ammonium but primarily limited by mesophyll conductance in all other treatment with the exception of seedlings treated with nitrate under eCO_2_ where it was limited almost equally by mesophyll conductance and biochemistry (**Figure 6A**). In contrast, the photosynthesis of boxelder maple was mainly limited by biochemical and *g_s_* (**Figure 6B**), particularly in the combination of nitrate and eCO_2_ (**Figure 6B**).

### 3.5 Adaptation to CO_2_ and N form

**Figure 7:**
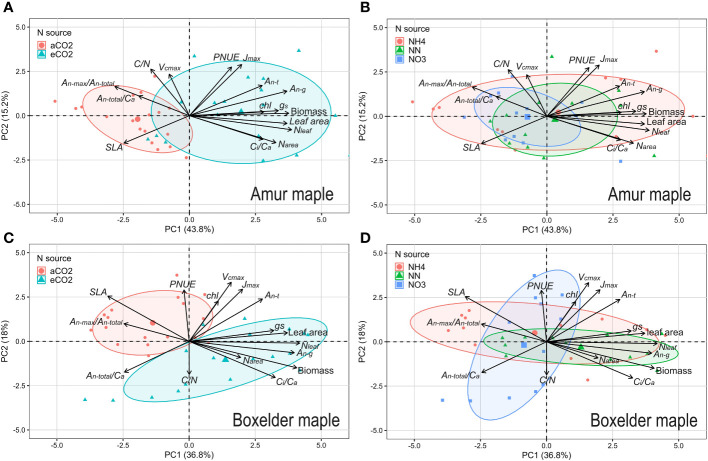
Principal Component Analysis (PCA) on growth and photosynthetic parameters in amur maple exposed to CO_2_ **(A)** and N resource **(B)** treatments and in boxelder maple **(C, D)**. The arrows point to near overlap, vertical, and reverse, which represent positive, no, and negative correlations between these parameters respectively. aCO_2_: ambient CO_2_ (400 µmol mol^−1^); eCO_2_: elevated CO_2_ (800 µmol mol^−1^); NH4: fertilized 10 mM (NH_4_)_2_SO_4_; NN: fertilized 10 mM N from 5 mM (NH_4_)_2_SO_4_ and 5 mM NaNO_3_; NO3: fertilized 10 mM NaNO_3_. *J_max_*: maximum of photosynthetic electron transport rate; *PNUE*: photosynthesis nitrogen use efficiency; *A_n-t_*: net photosynthesis rate at transition point (*C_i-t_*, *A_n-t_*) between Rubisco limitation and RuBP regeneration limitation based on *A/C_i_* curve; *A_n-g_*: net photosynthesis rate at a growth [CO_2_] which eCO_2_ at 800 µmol mol^−1^ and aCO_2_ at 400 µmol mol^−1^; *g_s_*: stomatal conductance; *N_leaf_*: total N of the whole-plant leaf; *Chl*: leaf chlorophyll concentration; *C_i_*/*C_a_*: the ratio of *C_i_* and *C_a_*; *N_area_*: leaf N per unit area; *SLA*: specific leaf area; *A_n-max_*/*A_n-total_*: the ratio of photosynthetic rate of saturation light at 400 µmol mol^−1^ (*A_n-max_*) and the y-intercept of *A_n_* vs. *C_i_* fitting line (*A_n-total_*) from light response curve database; *A_n-total/_C_a_*: the slope of *A_n_* vs. *C_i_* fitting line from light response curve database; C/N: leaf carbon and nitrogen ratio; *V_cmax_*: maximum rate of ribulose-1,5-bisphosphate carboxylation; See **Supplementary Table S1** for other explanations.

PCA showed an interesting result that the photosynthetic capacity parameters (*V_cmax_*, *J_max_*) did not orient to the ellipse of aCO_2_ in the two species (**Figures 7A, C**). Seedlings grown under the eCO_2_ were grouped into the ellipses with growth parameters (biomass and leaf area) and *A_n-g_* (**Figures 7A, C**). It is also interesting to note that *N_area_* and *N_leaf_* clustered in the eCO_2_ ellipse in amur maple (**Figure 7A**) while C/N clustered in the eCO_2_ ellipse in boxelder maple (**Figure 7B**). Different N forms had no obvious effect on the response to CO_2_ in amur maple since the three ellipses largely overlapped (**Figure 7B**). In boxelder maple, in contrast, nitrate clustered with *N_mass_* and *A_n-total_*/*C_a_*, while the mixed N treatment was clustered with growth parameters (**Figure 7D**). It is worth noting that *A_n-max_*/*A_n-total_* from lrc was negatively correlated with the growth parameters in both tree species at about 180-degree angles (**Figures 7A–D**), and a similar effect was found in *SLA* in boxelder maple (**Figures 7C, D**).

**Figure 8:**
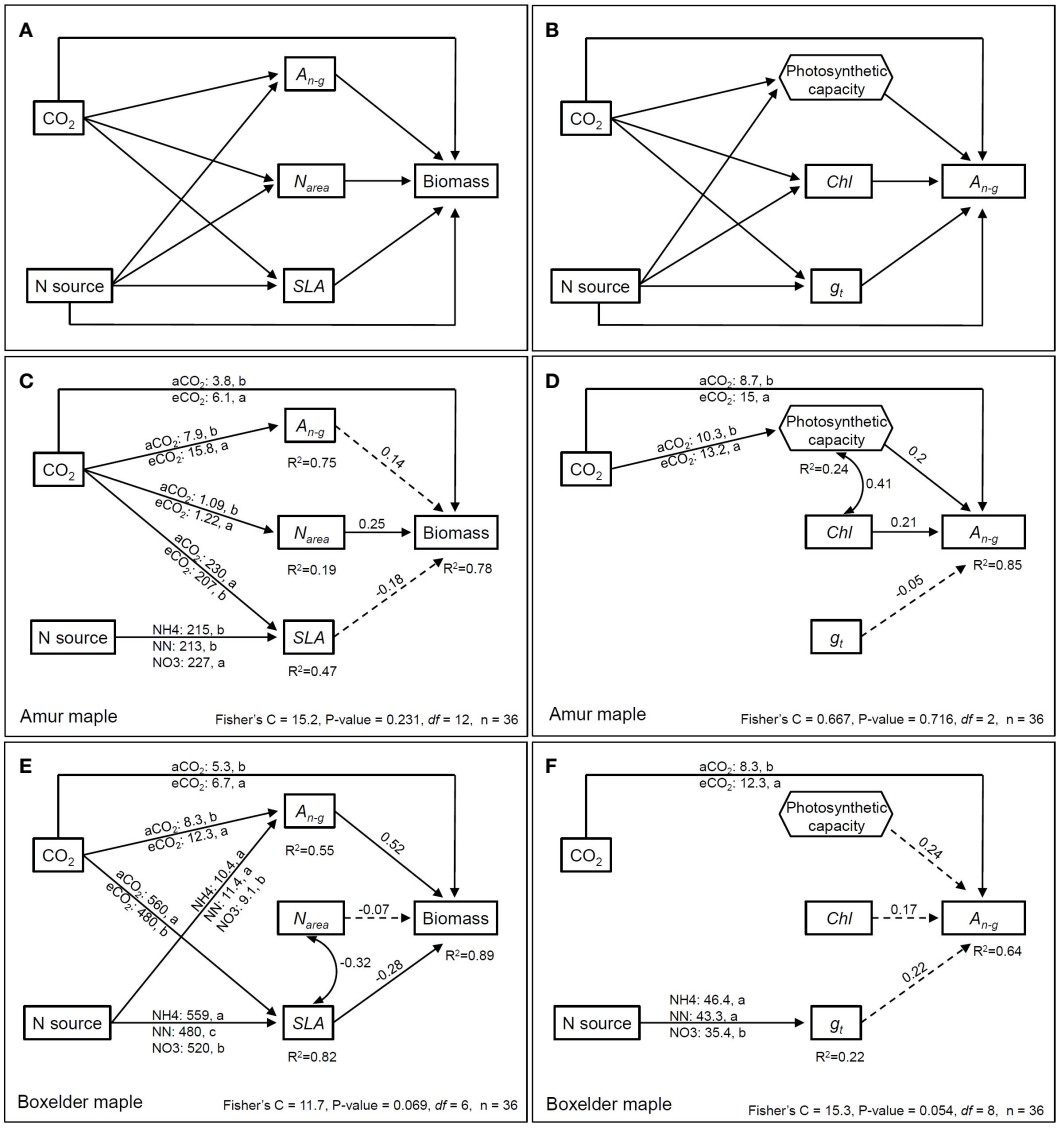
The *priori* (showing all tested paths) piecewise structural equation model (pSEM) relating to direct and indirect effects of CO_2_ and N form on biomass **(A)** and *A_n-g_***(B)**. Two CO_2_ treatments (ambient CO_2_: 400 µmol mol^−1^ and elevated CO_2_: 800 µmol mol^−1^) and N source (10 mM N source by three forms: NH_4_^+^, NH_4_^+^+NO_3_^-^ in 1:1, NO_3_^-^) are treated as categorical variables. *A_n-g_*: net photosynthesis rate at a growth [CO_2_] which eCO_2_ at 800 µmol mol^−1^ and aCO_2_ at 400 µmol mol^−1^; *N_area_*: leaf N per unit area; *SLA*: specific leaf area; *Chl*: leaf chlorophyll concentration; *g_t_*: total conductance to CO_2_ between the leaf surface and carboxylation sites (1/_gt_ = 1/*g_s_* + 1/*g_m_*). Multiple regression from *V_cmax_* and *J_max_* to *A_n-g_* was used to construct photosynthetic capacity as a component variable (hexagonal frame). The SEM (using psem function in piecewiseSEM package R) of biomass and *A_n-g_* in amur maple are in **(C, D)**, while that of boxelder maple are in **(E, F)**. Arrows mean the directional influence between the variables and the solid lines represent significant relationships, and the dashed lines refer to nonsignificant relationships (*P* > 0.05). The numbers on top of the arrows represent the standardized path coefficients (for continuous variables), and the numbers under the box with R^2^ refer to the degree of variation of the variable interpreted by all paths. Curved double arrows represent a significant correlation between variables (with correlation coefficient). The values on the line from the categorical variable indicated the continuous variable estimated marginal means by the treatment levels.

We constructed SEM (Structural Equation Model) to evaluate the direct/indirect effects on biomass and *A_n-g_* by the two treatments (CO_2_ and N) as categorical variables using piecewiseSEM package in R (**Figures 8A, B**). The results showed that CO_2_ treatment had direct effects on the biomass of both tree species (**Figures 8C, E**), indirect effects on the biomass through *N_area_* in amur maple (**Figure 8C**), and indirect effects on the biomass through *A_n-g_* and *SLA* in boxelder maple (**Figure 8E**). N forms had a significant effect on the *SLA* in amur maple, but its impact on biomass did not reach a significant level (**Figure 8C**). However, N treatment had a significant indirect effect on biomass through *A_n-g_* and *SLA* in boxelder maple (**Figure 8E**).

CO_2_ had a significant direct effect on the *A_n-g_* of both maple (**Figures 8D, F**), and a significant indirect effect on the biomass through photosynthesis in amur maple (**Figure 8D**). No significant effect of N form was observed on *A_n-g_* and related parameters in amur maple (**Figure 8D**, not shown). In boxelder maple, N treatment affected total CO_2_ conductance, but had no significant effect on *A_n-g_* (**Figure 8F**).

## 4 Discussion

### 4.1 Adaptation to elevated CO_2_

Elevated CO_2_ and associated climate change affect plant growth and distribution through via influencing physiological processes (Lauriks et al., 2022; Ma et al., 2023). Elevated CO_2_ usually causes photosynthetic adaptation, which is typically manifested by decreases in photosynthetic capacity (*V_cmax_*, *J_max_*), *N_area_* and *g_s_* (Hao et al., 2023), but promotes biomass production and photosynthetic rate (Dusenge et al., 2020; Tcherkez et al., 2020). However, there was no reduction in photosynthetic capacity or *N_area_* observed in either species in this study. To the contrary, eCO_2_ increased *V_cmax_* and *J_max_* in seedlings fertilized with ammonium. The photosynthetic downregulation induced by eCO_2_ is generally related to the dilution of leaf N and reduction in Rubisco as a result of increased carbohydrate production and growth (Dusenge et al., 2019).

Our PCA analysis shows that under aCO_2_ treatment, both amur maple and boxelder maple converged on the ellipse in the direction of *A_n-max_*/*A_n-total_*, (the ratio of *A_n-max_* and *A_n-total_* from lrc, see **Figure 1B** and **Supplementary Table S1**), and that *A_n-max_*/*A_n-total_* was always inversely proportional to *C_i_*/*C_a_*. From **Figure 1B**, *A_n-total_*/*C_a_* = *A_n-max_*_/_(*C_a_* - *C_i_*), so *A_n-max_*/*A_n-total_* = (*C_a_* - *C_i_*)/*C_a_*, which means that *A_n-max_*/*A_n-total_* + *C_i_*/*C_a_* = 1. This seems to demonstrate an intrinsic inverse relationship between *A_n-max_*/*A_n-total_* and *C_i_*/*C_a_*. Elevated CO_2_ reduced *A_n-max_*/*A_n-total_*, but increased *C_i_*/*C_a_* in both species (**Supplementary Tables S3**, **S4**). These results indicated that the photosynthetic adaptation processes in two maple species appeared diverse and highly plastic (Moejes et al., 2017).

The SEM revealed that eCO_2_ had positive effects on the biomass and *A_n-g_* of both species, but only the effect on *A_n-g_* in boxelder maple was statistically significant. CO_2_ is considered as a signal to control stomatal movement and growth metabolisms (Hao et al., 2023). Our results show that the biomass of both maple species was closely related to *A_n-g_* and CO_2_ in the absence of other stresses.

### 4.2 Biochemical and CO_2_ diffusion limitations to photosynthesis

This study shows that an important effect of eCO_2_ is that it shifted the biochemical limitation of photosynthesis from Rubisco carboxylation to RuBP regeneration, i.e., from *A_n-g_* < *A_n-t_* under aCO_2_ to *A_n-g_* > *A_n-t_* under eCO_2_ (Wang et al., 2022). This result suggests that under the condition of the projected future climate, photosynthesis may be mainly limited by *J_max_* rather than *V_cmax_* (Smith and Keenan, 2020). It should be noted that when photosynthesis is limited by *J_max_*, the sensitivity of *A_n_* to *C_i_* variation is reduced (Dusenge et al., 2019). The result that *C_i_*/*C_a_* ratio was higher under eCO_2_ than under aCO_2_ suggests that photosynthesis was less limited by CO_2_ diffusion in the two species grown under eCO_2_ (Lamba et al., 2018).

Photosynthetic capacity represents the CO_2_ fixation potential of leaves and does not represent the photosynthetic rate under actual growth conditions (Wang et al., 2022), since the net photosynthetic rate of C3 plants is also affected by substrate CO_2_ supply (Stefanski et al., 2020). For example, the photosynthetic capacity of amur maple treated with ammonium under aCO_2_ was maintained at a high level, while the actual *A_n_* was the lowest, which was obviously limited by low CO_2_ supply and low stomatal conductance (**Table 2**, **Figure 3A**). Our results support the theory that higher photosynthetic capacity does not always translate into higher *A_n_* (Xu et al., 2020).

The SEM showed that the resistance to CO_2_ diffusion was not a primary driver for differences in *A_n_* between treatments (**Figures 8D, F**) and the relationship appeared to be opposite between the two maple species. The resistance to CO_2_ diffusion mainly includes *g_s_* and *g_m_* and has a significant effect on photosynthesis (Grassi and Magnani, 2005). However, using *g_t_* [*g_t_* = *g_s_* * *g_m_*/(*g_s_* + *g_m_*)] to express the overall conductivity of CO_2_ seems to blur the individual effect of *g_s_* or *g_m_* (Grassi and Magnani, 2005). Although *g_s_* and *g_m_* jointly describe the diffusion of CO_2_ from leaf surface to carboxylation sites, they are independent processes with different regulatory mechanisms and physiological significance. The *g_s_* may be closely related to water status (Stefanski et al., 2020), while *g_m_* integrates biochemical and physical factors of intercellular CO_2_ diffusion (Berghuijs et al., 2016).

### 4.3 N forms and partitioning effects

It is believed that eCO_2_ is beneficial to plants that prefer ammonium over nitrate (Andrews et al., 2019; Dusenge et al., 2019). Our results show that ammonium fertilization led to the highest while nitrate resulted in the lowest photosynthesis and growth among the three fertilization treatments under eCO_2_. This result may be related to the lower energy consumption and higher metabolic efficiency of ammonium compared with nitrate (Bloom, 2015; Rubio-Asensio and Bloom, 2017). However, the accumulation of more ammonium in cells may cause ammonium toxicity, resulting in the decrease of *g_s_* and *A_n_*, chlorosis and growth inhibition (Vega-Mas et al., 2017). Our results that *Chl* and *g_s_* were lowest in seedlings fertilized with ammonium under aCO_2_ may indicate a toxic effect of ammonium to those trees. Ammonium toxicity is attributed to excessive ammonium ions that lead to cell ion disturbances, the depletion of organic acid, and acid stress caused by the proton’s mass formation (Hachiya et al., 2021). The eCO_2_ can promote the synthesis of organic acids by providing the carbon framework and alleviating ammonium toxicity (Vega-Mas et al., 2017; La Peña et al., 2022). Our results showed that ammonium might be beneficial to the growth of the two maple species in the future when CO_2_ is elevated. However, the mechanism of this synergistic effect of eCO_2_ and ammonium needs further study.

It is common in C3 plants that eCO_2_ inhibits the assimilation of nitrate (Rachmilevitch et al., 2004). The reason may be that eCO_2_ inhibits photorespiration, and nitrate assimilation depends on photorespiration-related reductants (Ainsworth and Long, 2021). Furthermore, eCO_2_ may also inhibit the activity of nitrate reductase (Wujeska-Klause et al., 2019). The mixed nitrate and ammonium treatment appeared more beneficial to the growth of boxelder maple than the application of only nitrate, this suggests that ammonium and nitrate may have some interactive effects on boxelder maple. Notably, N allocation may also play an important role in the regulation of photosynthesis (Wang et al., 2021). Amur maple treated by aNH4 and eNH4 had equivalent N allocation to photosynthesis (**Figure 4A**), but the photosynthetic rate and biomass in the latter treatment were more than twice that of the former. This was probably because the N in the eNH4 treatment was distributed more evenly to different components such as *N_et_*, *N_lc_* and *N_resp_*, than in the aNH4 treatment where there was a slight greater N allocation to *N_cb_*.

### 4.4 Plasticity and leaf morphological traits

Invasive plants generally have higher plasticity because they exhibit favorable phenotypes and robust adaptability in response to changes in environmental conditions (Godoy et al., 2012; Liu et al., 2017). Plasticity is reflected in the trade-offs between leaf structure and physiological progress described in the leaf economic spectrum (Liu et al., 2022), and in the optimal combination of key leaf traits *SLA*, *N_area_* and *A_n_* (Onoda et al., 2017). Successful invasive tree species often show higher *SLA* (lower leaf structure cost) and higher *N_area_* (higher nutrient resources), which is often associated with higher efficiency of resource acquisition, higher photosynthetic rates and faster investment returns (Xiong and Flexas, 2018). Our results show that both amur maple and boxelder maple exposed to eCO_2_ increased leaf construction cost (*SLA* lowered) and had higher *N_area_*. The high phenotypic plasticity may further facilitate the spread of specific invasive species in the future when the atmospheric CO_2_ elevation continues (Liu et al., 2017).

### 4.5 Adaptation strategy of amur maple and boxelder maple

Despite displaying comparable reactions to elevated CO_2_ and different nitrogen sources in terms of biomass and leaf nitrogen allocation, Amur maple and boxelder maple seem to have adopted contrasting adaptation strategies for future climate change. Amur maple appears to prioritize maximizing photosynthetic capacity per unit area of leaves, whereas boxelder maple seems to excel in increasing biomass through adaptations in leaf morphology and nitrogen utilization efficiency (**Supplementary Figures 3S, C**). This trend is also evident in relative photosynthetic limitation (**Figure 6**) and structural equation models (**Figures 8D, F**).

Our results provide further support for the conclusions of previous studies that the great plasticity has facilitated the successful invasion of boxelder maple into Europe, Asia and South America (Porté et al., 2011; McEvoy et al., 2022). A study in Lithuania finds that boxelder maple facilitates invasion by increasing the rate of foliar decomposition and nutrient cycling (Manusadžianas et al., 2014). Our results showed that the C/N and *N_leaf_* of boxelder maple were significantly higher than those of amur maple, suggesting that boxelder maple may speed soil nutrient cycling and the invasion of other species (Lee et al., 2017). A well-known paradox is that invasive plants reduce biodiversity but increase plant productivity (Rout and Callaway, 2009). A recent genomic comparison has found that boxelder maple has a smaller genome with recent gene family evolution which might be related to its robust adaptability (McEvoy et al., 2022). Even though the two maple trees have chosen different adaptation strategies for the future climate, they have both demonstrated a favorable reaction to the elevated CO2. It is important to note that the boxelder maple’s strategy of optimizing leaf morphology and nitrogen utilization efficiency not only showcases its impressive phenotypic plasticity, but also enhances its invasive potential by facilitating nutrient cycling between leaves and soil.

## 5 Conclusions

Invasive tree species generally have greater plasticity and more efficient utilization of carbon and nitrogen sources in the environment. We found that amur maple and boxelder maple both showed strong plasticity in response to eCO_2_ and variation in N sources. Furthermore, they seem to exhibit a coordinated response to the two treatment factors. Ammonium was not conducive to the growth and physiology of both species under current CO_2_ but significantly improved their performance under the elevated CO_2_. However, the effects of nitrate were the opposite. Boxelder maple seems to have stronger adaptability to future climate change than Amur maple. Because boxelder maple invests more N in its leaves and has a larger specific leaf area, indicating that boxelder maple could promote soil nutrient cycling and ecosystem function. We conclude that increases in soil ammonium will be beneficial to the plasticity and adaptation of amur maple and boxelder maple in the future as atmospheric CO_2_ continues to rise.

## Data availability statement

The raw data supporting the conclusions of this article will be made available by the authors, without undue reservation.

## Author contributions

LW: Data curation, Formal analysis, Investigation, Writing – original draft, Writing – review & editing. Q-LD: Conceptualization, Funding acquisition, Investigation, Methodology, Project administration, Resources, Software, Supervision, Validation, Writing – review & editing.

## Supplementary material

Supplementary Table 1 Definition of acronyms.

Supplementary Table 2 ANOVA *P*-values of CO_2_ and N source effects on photosynthetic parameters shown in **Figure 4**. Note: CO_2_ treatment includes ambient CO_2_ (400 µmol mol^−1^) and elevated CO_2_ (800 µmol mol^−1^) and N treated with 10 mM N by three forms: NH_4_^+^, NH_4_^+^+NO_3_^-^ in 1:1, NO_3_^-^).

Supplementary Table 3 Photosynthetic parameters (mean ± SEM) derived from photosynthetic light response curves (LRC) in amur maple seedlings grown under different CO_2_ and N forms. Note: each value represents mean ± SE (n=6). Two-way ANOVA was performed to analyze CO_2_ and nitrogen (N) as well as their interactive effects (CO_2_: N). Significant effects (*P* ≤ 0.05) are shown in bold and “:” indicated interaction. Different letters within the same column indicated statistically significant differences between treatments (Tukey *post hoc* test, P<0.05). aCO_2_: ambient CO_2_ (400 µmol mol^−1^); eCO_2_: elevated CO_2_ (800 µmol mol^−1^); NH4: fertilized 10 mM (NH_4_)_2_SO_4_; NN: fertilized 10 mM N with 5 mM (NH_4_)_2_SO_4_ and 5 mM NaNO_3_; NO3: fertilized 10 mM NaNO_3_. Abbreviations are provided in **Supplementary Table S1**.

Supplementary Table 4 Photosynthetic parameters (mean ± SEM) derived from photosynthetic light response curves (LRC) in boxelder maple seedlings grown under different CO_2_ and N forms. Note: each value represents mean ± SE (n=6). Two-way ANOVA was performed to analyze CO_2_ and nitrogen (N) as well as their interactive effects (CO_2_: N). Significant effects (*P* ≤ 0.05) are shown in bold and “:” indicated interaction. Different letters within the same column indicated statistically significant differences between treatments (Tukey *post hoc* test, P<0.05). aCO_2_: ambient CO_2_ (400 µmol mol^−1^); eCO_2_: elevated CO_2_ (800 µmol mol^−1^); NH4: fertilized 10 mM (NH_4_)_2_SO_4_; NN: fertilized 10 mM N with 5 mM (NH_4_)_2_SO_4_ and 5 mM NaNO_3_; NO3: fertilized 10 mM NaNO_3_. Abbreviations are provided in **Supplementary Table S1**.

Supplementary Figure 1 CO_2_ concentration **(A)** and air temperature in **(B)** for the four climate-controlled glasshouses. The conditions were recorded 24 h a day every day. CO_2_ setting was 800 µmol mol^−1^ for the elevated and 400 µmol mol^−1^ for the ambient treatment. Day 1 indicates the beginning of the experiment and Day 120 indicates the completion of the experiment.

Supplementary Figure 2 Pictures of amur maple **(A)** and boxelder maple **(B)**) in different treatment combinations. “a-” means ambient CO2 (400 µmol mol−1) and “e-” means elevated CO2 (800 µmol mol−1) treatments. NH4: fertilized 10 mM (NH4)2SO4; NN: fertilized 10 mM N with 5 mM (NH4)2SO4 and 5 mM NaNO3; NO3: fertilized 10 mM NaNO3.

Supplementary Figure 3 Principal Component Analysis (PCA) on growth and photosynthetic parameters of seedlings exposed to CO_2_ **(A)** and N resource **(B)** and in two species **(C)**. The arrow direction of near overlap, vertical, and reverse, indicates positive correlation, no correlation, and negative correlation between these parameters, respectively. aCO_2_: ambient CO_2_ (400 µmol mol^−1^); eCO_2_: elevated CO_2_ (800 µmol mol^−1^); NH4: fertilized 10 mM (NH_4_)_2_SO_4_; NN: fertilized 10 mM N from 5 mM (NH_4_)_2_SO_4_ and 5 mM NaNO_3_; NO3: fertilized 10 mM NaNO_3_. *J_max_*: maximum of photosynthetic electron transport rate; *PNUE*: photosynthesis nitrogen use efficiency; *A_n-t_*: net photosynthesis rate at transition point (*C_i-t_*, *A_n-t_*) between Rubisco limitation and RuBP regeneration limitation based on *A/C_i_* curve; *A_n-g_*: net photosynthesis rate at a growth [CO_2_] which eCO_2_ at 800 µmol mol^−1^ and aCO_2_ at 400 µmol mol^−1^; *g_s_*: stomatal conductance; *N_leaf_*: total N of the whole-plant leaf; *Chl*: leaf chlorophyll concentration; *C_i_*/*C_a_*: the ratio of *C_i_* and *C_a_*; *N_area_*: leaf N per unit area; *N_mass_*: leaf N concentration; *SLA*: specific leaf area; *Γ_ACi_*: CO_2_ compensation point from *A*/*C_i_* curve; *A_n-max_*/*A_n-total_*: the ratio of photosynthetic rate of saturation light at 400 µmol mol^−1^ (*A_n-max_*) and the y-intercept of *A_n_* vs. *C_i_* fitting line (*A_n-total_*) from light response curve database; △*A_n_*/△*C_i-lrc_*: the slope of *A_n_* vs. *C_i_* fitting line from light response curve database; C/N: leaf carbon and nitrogen ratio; *V_cmax_*: maximum rate of ribulose-1,5-bisphosphate carboxylation; *ACE*: apparent carboxylation efficiency. See **Supplementary Table S1** for other explanations.
