# Supplementary material for: Elevated CO2 and ammonium nitrogen promoted the plasticity of two maple in great lakes region by adjusting photosynthetic adaptation
Source: Front Plant Sci. 2024 Apr 9;15:1367535. doi: 10.3389/fpls.2024.1367535 (PMC11035798; doi:10.3389/fpls.2024.1367535)
Supplement: Supplementary Table 1 — Definition of acronyms. [file DataSheet_1.docx]

**Supplementary materials**

**Table S1** Definition of acronyms

| Acronyms | Definitions | Unit |
| --- | --- | --- |
| [CO_2_] | CO_2_ concentration | µmol mol^−1^ |
| aNH4 | Treatment with ambient [CO_2_] 400 µmol mol^−1^ and 10 mM N fertilizer with (NH_4_)_2_SO_4_ | - |
| aNN | Treatment with ambient [CO_2_] 400 µmol mol^−1^ and 10 mM N fertilizer with 5mM (NH_4_)_2_SO_4_ and with 5mM NaNO_3_ | - |
| aNO3 | Treatment with ambient [CO_2_] 400 µmol mol^−1^ and 10 mM N fertilizer with NaNO_3_ | - |
| am | Amur maple (*Acer ginnala* Maxim.) | - |
| *A/C_i_* | Net photosynthesis rate vs. CO_2_ response curve | *-* |
| *ACE* | Apparent carboxylation efficiency (the initial slope of *A*/*C_i_* curve) | *-* |
| *A_n_* | Net photosynthesis rate | µ mol m^-2^ s^-1^ |
| *A_n-g_* | *A_n_* at a growth [CO_2_] which eCO_2_ at 800 µmol mol^−1^ and aCO_2_ at 400 µmol mol^−1^ | µmol mol^−1^ |
| *A_n-t_* | *A_n_* at the photosynthetic transition point between RuBP carboxylation to regeneration limitation | µ mol m^-2^ s^-1^ |
| *A_n-max_* | The photosynthetic rate of saturation light at 400 µmol mol^−1^ *C_a_* from lrc (Fig. 1a) | µ mol m^-2^ s^-1^ |
| *A_n-total_* | The y-intercept of *A_n_* vs. *C_i_* fitting line from the light response curve database (see Fig. 1b) | µ mol m^-2^ s^-1^ |
| *A_n-max_/A_n-total_* | The ratio of *A_n-max_* and *A_n-total_* from lrc | - |
| *A_n-tatol_ /C_a_* | The slope of *A_n_* vs. *C_i_* fitting line from the light response curve database (see Fig. 1b) |  |
| *AQY* | Apparent quantum yield (the initial slope of lrc) | - |
| bm | Boxelder maple (*Acer negundo* L.) | - |
| *C_a_* | Ambient CO_2_ concentration | µmol mol^−1^ |
| *Chl* | Leaf chlorophyll concentration | mg m^-2^ |
| *C_i_* | Intercellular CO_2_ concentration | µmol mol^−1^ |
| *C_i_** | Intercellular CO_2_ compensation point | µmol mol^−1^ |
| *C_i_/C_a_* | The ratio of *C_i_* and *C_a_* |  |
| *C_i-g_* | *C_i_* at growth [CO_2_] which Eco_2_ at 800 µmol mol^−1^ and Aco_2_ at 400 µmol mol^−1^ | µmol mol^−1^ |
| *C_i-t_* | *C_i_* at the transition point between RuBP carboxylation to regeneration limitation | µmol mol^−1^ |
| *C/N* | Leaf carbon and nitrogen ratio | - |
| eNH4 | Treatment with elevated [CO_2_] 800 µmol mol^−1^ and 10 Mm N fertilizer by (NH_4_)_2_SO_4_ | - |
| eNN | Treatment with elevated [CO_2_] 800 µmol mol^−1^ and 10 Mm N fertilizer by 5Mm (NH_4_)_2_SO_4_ and by 5Mm NaNO_3_ | - |
| eNO3 | Treatment with elevated [CO_2_] 800 µmol mol^−1^ and 10 Mm N fertilizer by NaNO_3_ | - |
| *_Ə_A*/*_Ə_C_c_* | The initial slope of *A_n_* versus *C_c_* curve | - |
| *Γ_ACi_* | CO_2_ compensation point from *A/C_i_* curve | µmol mol^−1^ |
| *g_m_* | Mesophyll conductance | mol m^-2^ s^-1^ |
| *g_s_* | Stomatal conductance | mmol m^-2^ s^-1^ |
| *g_t_* | Total conductance to CO_2_ between the leaf surface and carboxylation sites (1/*g_t_* = 1/*g_s_* + 1/*g_m_*) | mmol m^-2^ s^-1^ |
| *J_max_* | Maximum photosynthetic electron transport rate | µ mol m^-2^ s^-1^ |
| lrc | Light response curve | - |
| *LCP* | Light intensity compensation point | µ mol m^-2^ s^-1^ |
| *l_b_* | Relative photosynthesis limitation due to biochemical capacity | - |
| *l_m_* | Relative photosynthesis limitation due to *g_m_* | - |
| *l_s_* | Relative photosynthesis limitation due to *g_s_* | - |
| *N_cb_* | Leaf N partitioning into carboxylation | - |
| *N_et_* | Leaf N partitioning into electron transfer | - |
| *N_lc_* | Leaf N partitioning into light capture systems | - |
| *N_resp_* | Leaf N partitioning into respiratory | - |
| *N_area_* | Leaf nitrogen (N) content based on leaf-area | g m^-2^ |
| *N_leaf_* | Total N of the whole-plant leaf (*N_mass_* x leaf mass) | mg plant^-1^ |
| PAR | Photosynthetically active radiation | µ mol m^-2^ s^-1^ |
| *PNUE* | Photosynthetic nitrogen uses efficiency (*A_n_* / *N_area_*) | µ mol CO_2_ g^-1^ N s^-1^ |
| *R_d_* | Daytime respiration | µ mol m^-2^ s^-1^ |
| *R_l_* | Photorespiration rate | µ mol m^-2^ s^-1^ |
| *R_t_* | Total leaf respiration under light | µ mol m^-2^ s^-1^ |
| *SLA* | Specific leaf area | cm^2^ g^-1^ |
| Theta (θ) | The curvature of the light response curve | - |
| *V_cmax_* | The maximum rate of Rubisco carboxylation | µ mol m^-2^ s^-1^ |

**Table S2** ANOVA *P*-values of CO_2_ and N source effects on photosynthetic parameters shown in Figure 4.

| Species | Treatments | *C_i-t_* | *A_n-t_* | *C_i-g_* | *A_n-g_* | *ACE* | *Γ_ACi_* |
| --- | --- | --- | --- | --- | --- | --- | --- |
| Amur maple | CO_2_ | 0.001 | 0.003 | **< 0.001** | < 0.001 | 0.146 | 0.001 |
| Amur maple | N | 0.342 | 0.765 | 0.775 | 0.614 | 0.082 | 0.004 |
| Amur maple | CO_2_ : N | **< 0.001** | **< 0.001** | 0.138 | **< 0.001** | **0.005** | **< 0.001** |
| Boxelder maple | CO_2_ | **< 0.001** | 0.505 | **< 0.001** | < 0.001 | 0.005 | 0.205 |
| Boxelder maple | N | 0.311 | 0.296 | 0.264 | 0.002 | 0.634 | 0.704 |
| Boxelder maple | CO_2_ : N | 0.109 | **0.001** | 0.822 | **< 0.001** | **< 0.001** | **0.006** |

Note: CO_2_ treatment includes ambient CO_2_ (400 µmol mol^−1^) and elevated CO_2_ (800 µmol mol^−1^) and N treated with 10 mM N by three forms: NH_4_^+^, NH_4_^+^+NO_3_^-^ in 1:1, NO_3_^-^).

**Table S3** Photosynthetic parameters (mean ± SEM) derived from photosynthetic light response curves (LRC) in amur maple seedlings grown under different CO_2_ and N forms.

| CO_2_ | N | Theta (θ) | *AQY* | *LCP* | *A_n-max_* | *A_n-max_/ A_n-total_* | *A_n-total_* /*C_a_* |
| --- | --- | --- | --- | --- | --- | --- | --- |
| aCO_2_ | NH4 | 0.88±0.04 ab | 0.039±0.009 a | 48.3±3.1 a | 7.8±1.1 b | 0.43±0.035 a | -0.026±0.006 a |
|  | NN | 0.9±0.03 ab | 0.028±0.003 b | 35.9±9 ab | 8.4±0.8 b | 0.41±0.047 a | -0.052±0.011 ab |
|  | NO3 | 0.85±0.06 b | 0.032±0.004 ab | 32.3±2.7 b | 9.2±0.8 b | 0.43±0.027 a | -0.049±0.006 ab |
| eCO_2_ | NH4 | 0.96±0.01 a | 0.033±0.002 ab | 45.5±2.7 ab | 11.9±0.5 a | 0.34±0.022 a | -0.081±0.007 b |
|  | NN | 0.94±0.01 ab | 0.032±0.003 ab | 41.7±5.2 ab | 9.4±1 ab | 0.37±0.036 a | -0.027±0.026 a |
|  | NO3 | 0.92±0.02 ab | 0.032±0.002 ab | 32.4±6.1 b | 8.2±1 b | 0.38±0.032 a | -0.052±0.011 ab |
| *P*-value | CO_2_ | **0.027** | 0.863 | 0.813 | 0.066 | 0.054 | 0.317 |
|  | N | 0.604 | 0.388 | **0.035** | 0.402 | 0.829 | 0.567 |
|  | CO_2_ : N | 0.856 | 0.539 | 0.715 | **0.027** | 0.756 | **0.018** |

Note: each value represents mean ± SE (n=6). Two-way ANOVA was performed to analyze CO_2_ and nitrogen (N) as well as their interactive effects (CO_2_ : N). Significant effects (*P* ≤ 0.05) are shown in bold and “:” indicated interaction. Different letters within the same column indicated statistically significant differences between treatments (Tukey post hoc test, P<0.05). aCO_2_: ambient CO_2_ (400 µmol mol^−1^); eCO_2_: elevated CO_2_ (800 µmol mol^−1^); NH4: fertilized 10 mM (NH_4_)_2_SO_4_; NN: fertilized 10 mM N with 5 mM (NH_4_)_2_SO_4_ and 5 mM NaNO_3_; NO3: fertilized 10 mM NaNO_3_. Abbreviations are provided in Table S1.

**Table S4** Photosynthetic parameters (mean ± SEM) derived from photosynthetic light response curves (LRC) in boxelder maple seedlings grown under different CO_2_ and N forms.

| CO_2_ | N | Theta (θ) | *AQY* | *LCP* | *A_n-max_* | *A_n-max_/ A_n-total_* | *△A_n-total_* /*C_a_* |
| --- | --- | --- | --- | --- | --- | --- | --- |
| aCO_2_ | NH4 | 0.97±0.009 ab | 0.047±0.0017 cd | 16.2±2.4 ab | 7.4±0.4 bc | 0.41±0.015 a | -0.04±0.001 a |
|  | NN | 0.96±0.011 ab | 0.043±0.0021 d | 19.6±2.3 a | 8.2±0.5 b | 0.4±0.009 ab | -0.049±0.004 ab |
|  | NO3 | 0.94±0.007 b | 0.049±0.0013 bc | 20.3±3.7 a | 10.2±0.4 a | 0.38±0.008 ab | -0.061±0.002 b |
| eCO_2_ | NH4 | 0.95±0.008 ab | 0.056±0.0006 a | 14.1±1.4 ab | 8.3±0.8 b | 0.35±0.013 b | -0.055±0.007 b |
|  | NN | 0.98±0.004 a | 0.053±0.0018 ab | 11±0.3 b | 8.1±0.2 b | 0.37±0.008 ab | -0.054±0.003 b |
|  | NO3 | 0.95±0.012 ab | 0.051±0.0016 bc | 10.7±2 b | 6.4±0.7 c | 0.37±0.019 ab | -0.04±0.004 a |
| *P*-value | CO_2_ | 0.717 | **< 0.001** | **< 0.001** | 0.028 | **0.002** | 0.932 |
|  | N | **0.041** | 0.117 | 0.989 | 0.683 | 0.79 | 0.694 |
|  | CO_2_ : N | 0.061 | 0.053 | 0.219 | **< 0.001** | 0.111 | **< 0.001** |

Note: each value represents mean ± SE (n=6). Two-way ANOVA was performed to analyze CO_2_ and nitrogen (N) as well as their interactive effects (CO_2_: N). Significant effects (*P* ≤ 0.05) are shown in bold and “:” indicated interaction. Different letters within the same column indicated statistically significant differences between treatments (Tukey post hoc test, P<0.05). aCO_2_: ambient CO_2_ (400 µmol mol^−1^); eCO_2_: elevated CO_2_ (800 µmol mol^−1^); NH4: fertilized 10 mM (NH_4_)_2_SO_4_; NN: fertilized 10 mM N with 5 mM (NH_4_)_2_SO_4_ and 5 mM NaNO_3_; NO3: fertilized 10 mM NaNO_3_. Abbreviations are provided in Table S1.


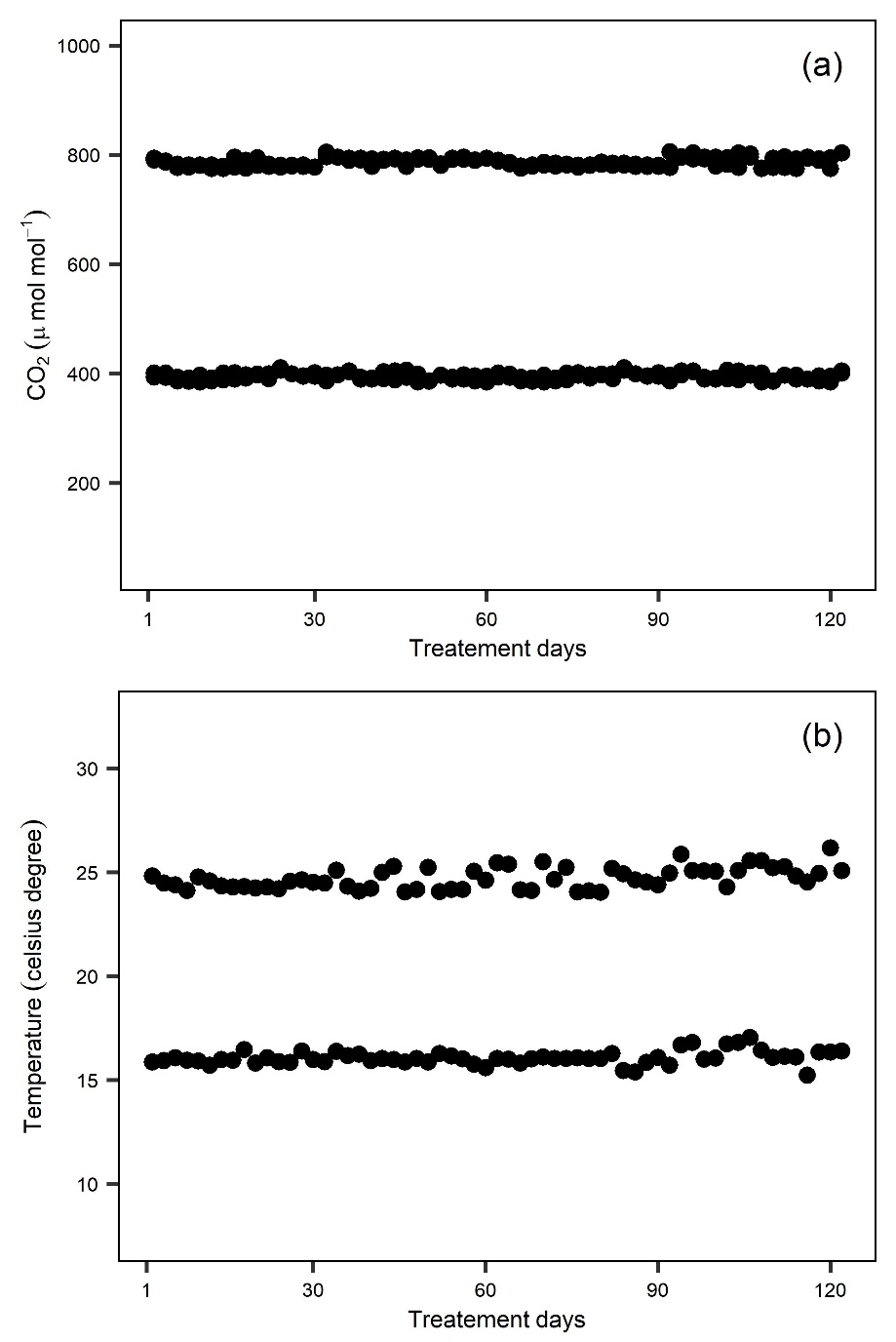


**Fig. S1** CO_2_ concentration (a) and air temperature in (b) for the four climate-controlled glasshouses. The conditions were recorded 24 h a day every day. CO_2_ setting was 800 µmol mol^−1^ for the elevated and 400 µmol mol^−1^ for the ambient treatment. Day 1 indicates the beginning of the experiment and Day 120 indicates the completion of the experiment.


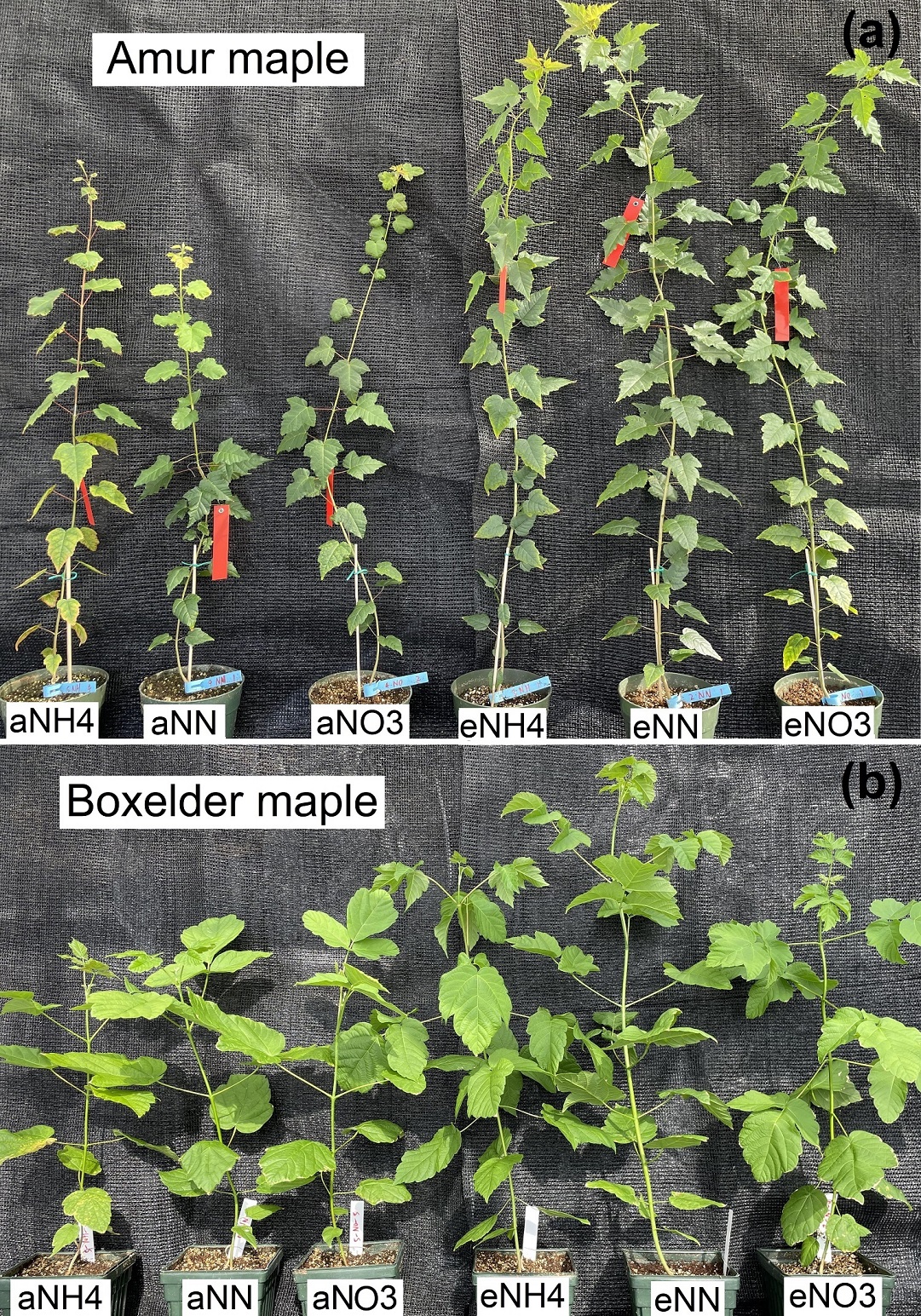


**Fig. S2** Pictures of amur maple (a) and boxelder maple (b)) in different treatment combinations. “a-” means ambient CO2 (400 µmol mol−1) and “e-” means elevated CO2 (800 µmol mol−1) treatments. NH4: fertilized 10 mM (NH4)2SO4; NN: fertilized 10 mM N with 5 mM (NH4)2SO4 and 5 mM NaNO3; NO3: fertilized 10 mM NaNO3.


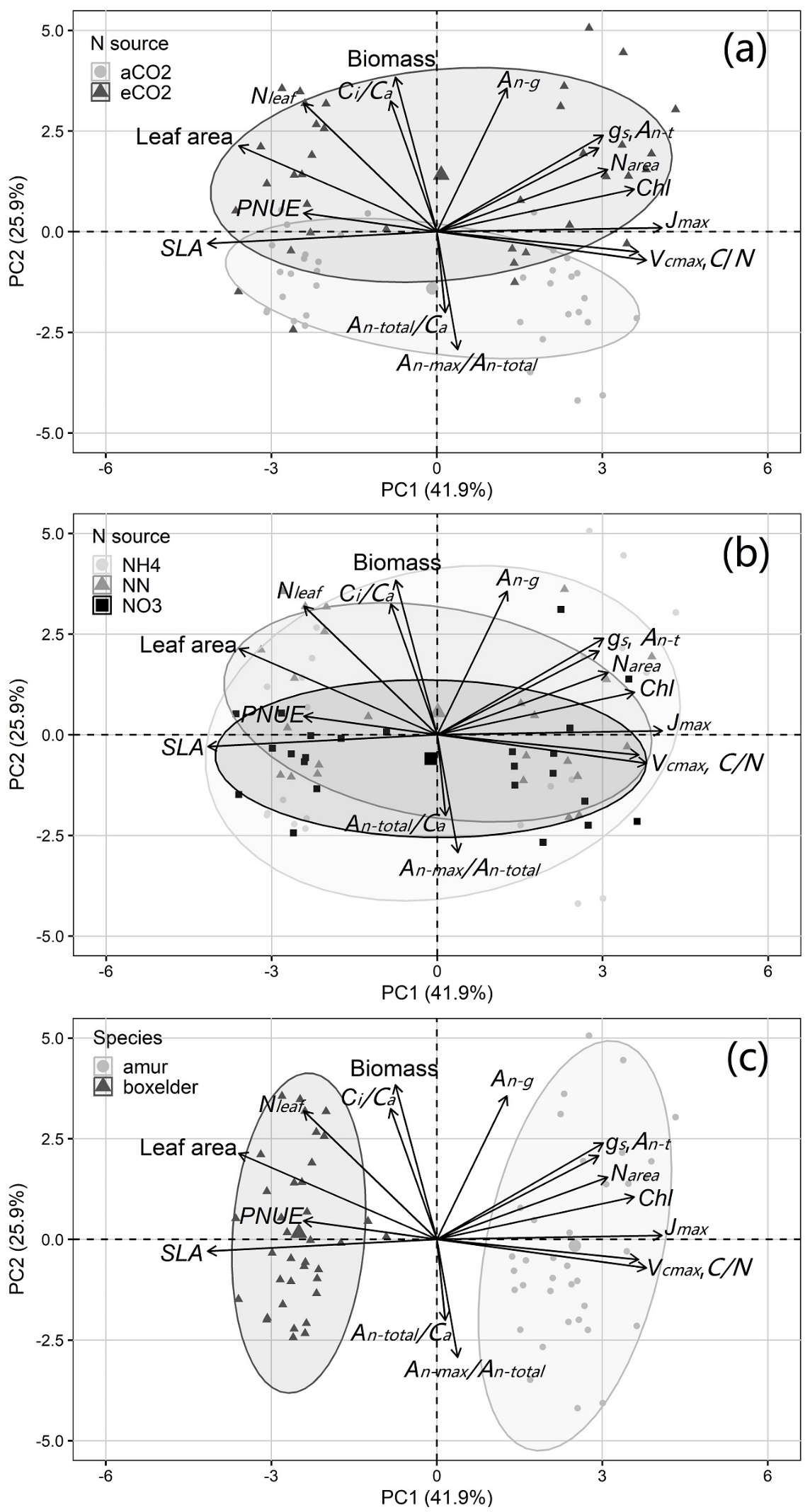


**Fig. S3** Principal Component Analysis (PCA) on growth and photosynthetic parameters of seedlings exposed to CO_2_ (a) and N resource (b) and in two species (c). The arrow direction of near overlap, vertical, and reverse, indicates positive correlation, no correlation, and negative correlation between these parameters, respectively. aCO_2_: ambient CO_2_ (400 µmol mol^−1^); eCO_2_: elevated CO_2_ (800 µmol mol^−1^); NH4: fertilized 10 mM (NH_4_)_2_SO_4_; NN: fertilized 10 mM N from 5 mM (NH_4_)_2_SO_4_ and 5 mM NaNO_3_; NO3: fertilized 10 mM NaNO_3_. *J_max_*: maximum of photosynthetic electron transport rate; *PNUE*: photosynthesis nitrogen use efficiency; *A_n-t_*: net photosynthesis rate at transition point (*C_i-t_*, *A_n-t_*) between Rubisco limitation and RuBP regeneration limitation based on *A/C_i_* curve; *A_n-g_*: net photosynthesis rate at a growth [CO_2_] which eCO_2_ at 800 µmol mol^−1^ and aCO_2_ at 400 µmol mol^−1^; *g_s_*: stomatal conductance; *N_leaf_*: total N of the whole-plant leaf; *Chl*: leaf chlorophyll concentration; *C_i_*/*C_a_*: the ratio of *C_i_* and *C_a_*; *N_area_*: leaf N per unit area; *N_mass_*: leaf N concentration; *SLA*: specific leaf area; *Γ_ACi_*: CO_2_ compensation point from *A*/*C_i_* curve; *A_n-max_*/*A_n-total_*: the ratio of photosynthetic rate of saturation light at 400 µmol mol^−1^ (*A_n-max_*) and the y-intercept of *A_n_* vs. *C_i_* fitting line (*A_n-total_*) from light response curve database; △*A_n_*/△*C_i-lrc_*: the slope of *A_n_* vs. *C_i_* fitting line from light response curve database; C/N: leaf carbon and nitrogen ratio; *V_cmax_*: maximum rate of ribulose-1,5-bisphosphate carboxylation; *ACE*: apparent carboxylation efficiency. See Table S1 for other explanations.
